# Supplementary figures and images for: Comprehensive metabolomics of Philippine Stichopus cf. horrens reveals diverse classes of valuable small molecules for biomedical applications
Source: PLoS One. 2023 Dec 6;18(12):e0294535. doi: 10.1371/journal.pone.0294535 (PMC10699614; doi:10.1371/journal.pone.0294535)

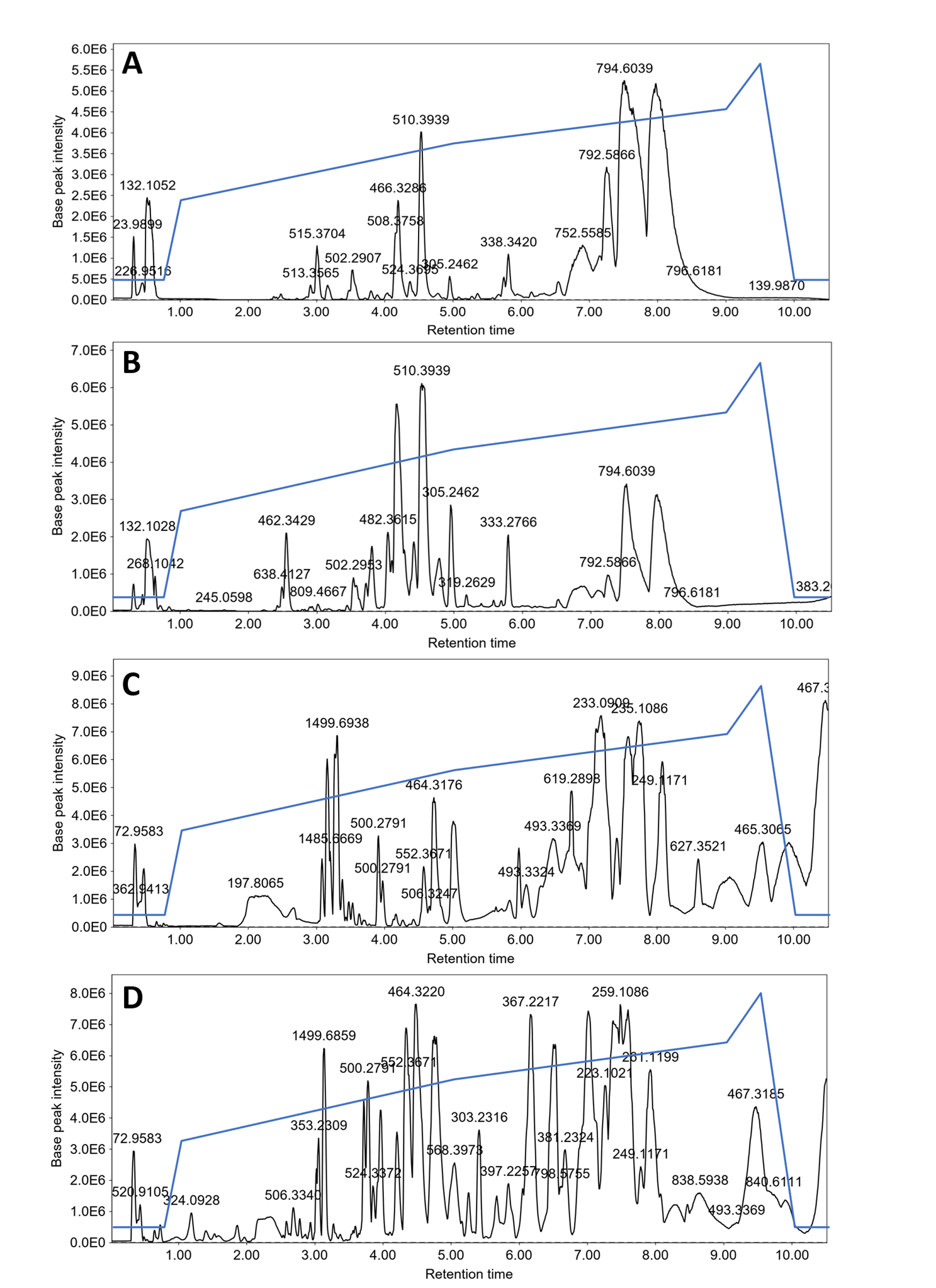

Supplement: S1 Fig — Metabolite fingerprints were obtained for the body wall and the viscera in the positive (A, B) and negative (C, D) mode respectively. (TIF) [file pone.0294535.s001.tif]

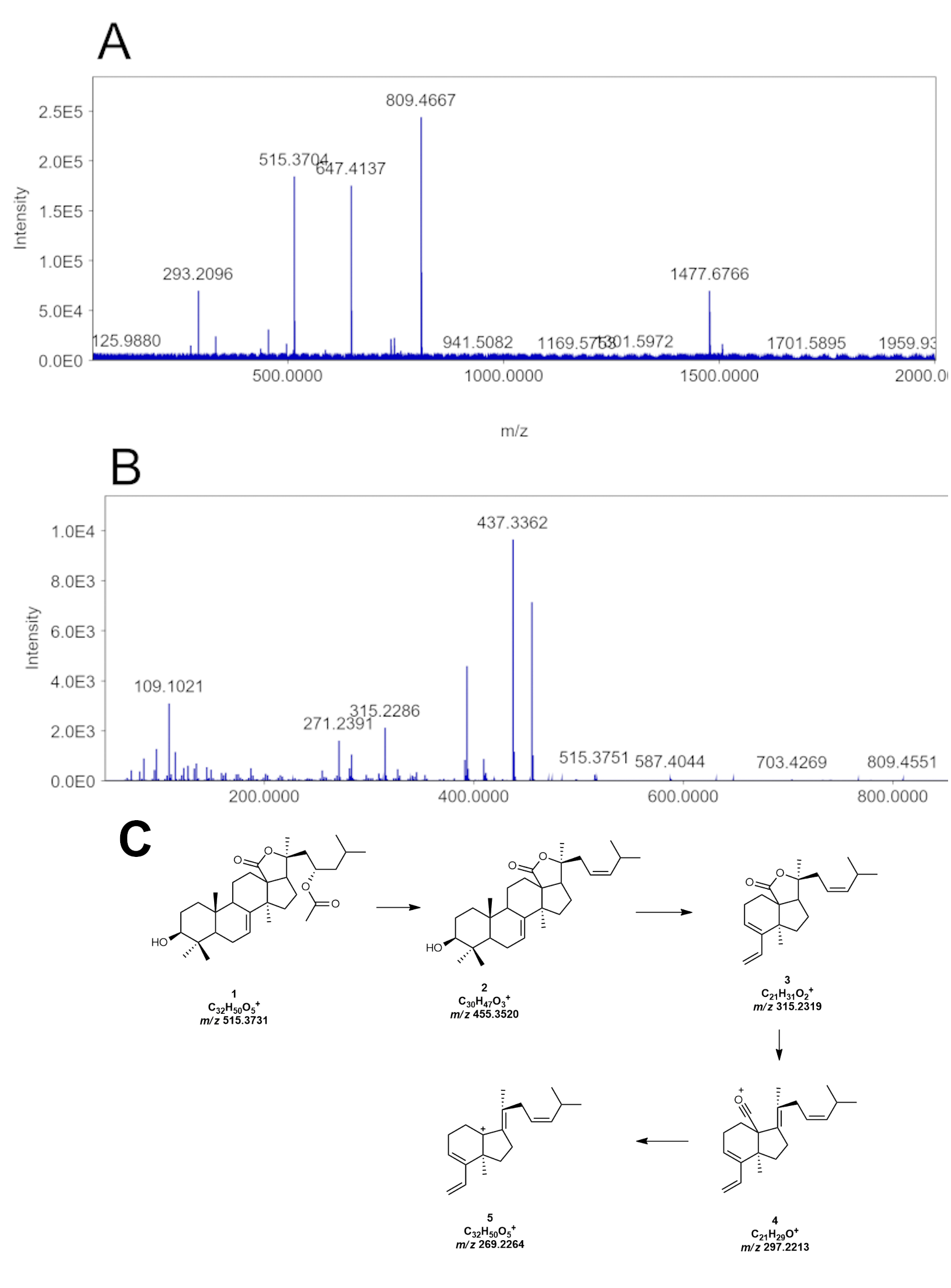

Supplement: S3 Fig — The MS1 and MS2 spectra are shown as (A) and (B) respectively, and the proposed reaction mechanism is shown in (C). Reactions proposed for the fragmentation are as follows: a) loss of the acetyl group (Δ60 Da), b) retro-Diels-Alder at the 2nd ring of the sapogenin, c) opening of the lactone ring followed by a subsequent loss of water (Δ18 Da), and d) decarbonylation (Δ28 Da). (TIF) [file pone.0294535.s003.tif]

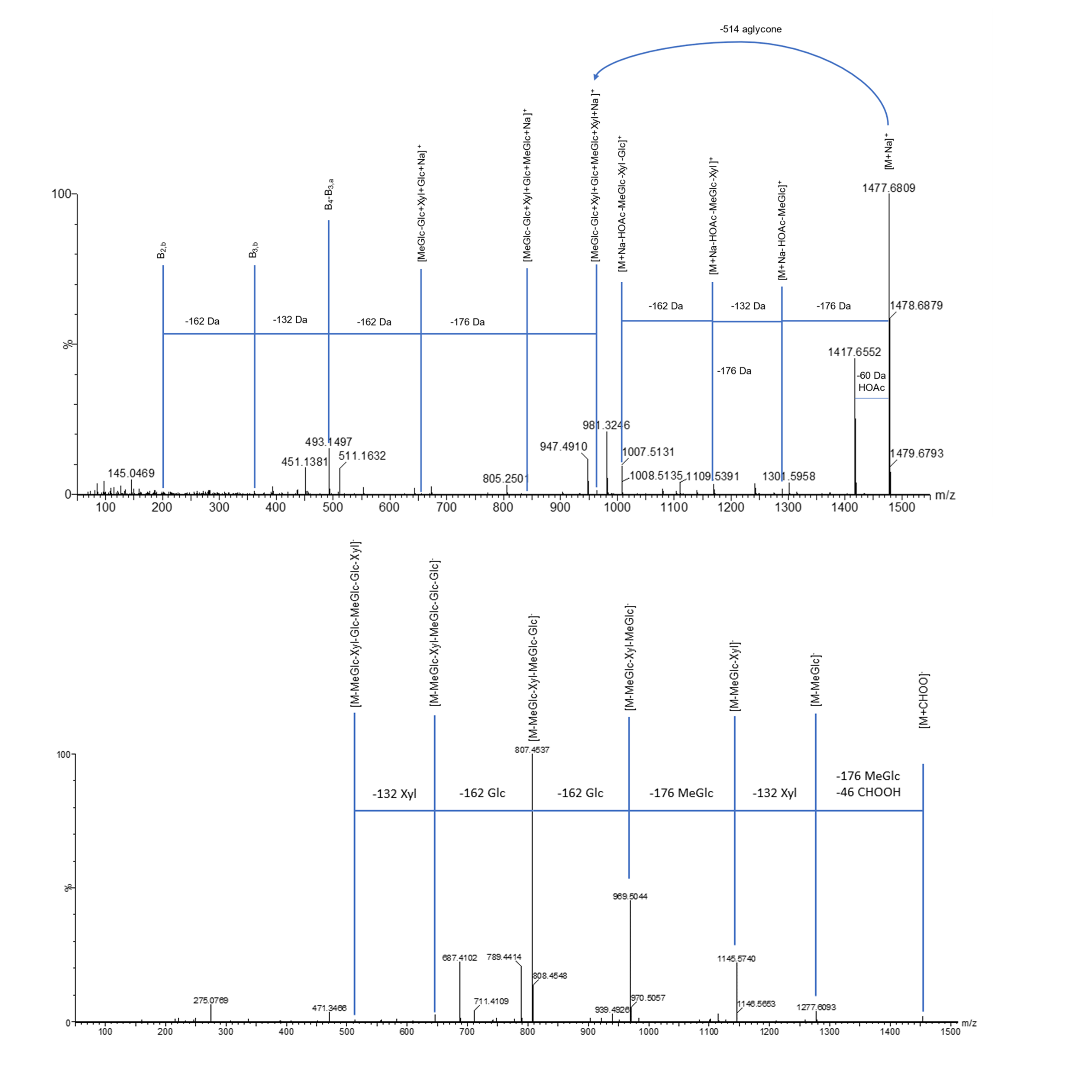

Supplement: S4 Fig — Parent ion in the positive mode is [M+Na]+ = 1477.6719 and the negative mode is [M+CHOO]- = 1499.6859. (TIF) [file pone.0294535.s004.tif]
