# Supplementary material for: Comprehensive metabolomics of Philippine Stichopus cf. horrens reveals diverse classes of valuable small molecules for biomedical applications
Source: PLoS One. 2023 Dec 6;18(12):e0294535. doi: 10.1371/journal.pone.0294535 (PMC10699614; doi:10.1371/journal.pone.0294535)
Supplement: S1 Table — (DOCX) [file pone.0294535.s006.docx]

**S1 Table. List of putatively identified primary metabolites from *S. cf. horrens*.**

|  | **Compound Name** | **tR**  **(mins.)** | **Major**  **Ion** | **Experimental**  **Mass** | **Theoretical**  **Mass** | **ppm**  **error** | **Cosine** | **Body Wall** | | | **Viscera** | | |
| --- | --- | --- | --- | --- | --- | --- | --- | --- | --- | --- | --- | --- | --- |
|  |  |  |  |  |  |  |  | **crude** | **iBOH** | **hex** | **crude** | **iBOH** | **hex** |
| 1 | N-Methylisoleucine | **0.46** | [M+H]+ | 146.1169 | 146.1176 | 4.52 | 0.97 |  |  |  |  |  |  |
| 2 | L-Carnitine | 0.49 | [M+H]+ | 162.1128 | 162.1125 | 2.04 | 0.94 |  |  |  |  |  |  |
| 3 | Adenosine | 0.65 | [M+H]+ | 268.1042 | 268.1046 | 1.49 | 0.97 |  |  |  |  |  |  |
| 4 | Adenine | 0.71 | [M+H]+ | 136.0617 | 136.0623 | 4.41 | 0.96 |  |  |  |  |  |  |
| 5 | Trytophan | 0.89 | [M+H]+ | 188.0709 | 188.0706 | 1.60 | 0.95 |  |  |  |  |  |  |
| 6 | Lauryldiethanolamine | 2.40 | [M+H]+ | 274.2735 | 274.2746 | 4.01 | 0.96 |  |  |  |  |  |  |
| 7 | Cholesterol | 10.23 | [M+H]+ | 369.3523 | 369.3521 | 0.54 | 0.83 |  |  |  |  |  |  |
| 8 | Inosine | 0.54 | [M-H]- | 267.0732 | 267.0735 | 1.09 | MN/FA |  |  |  |  |  |  |
