# Supplementary material for: Comprehensive metabolomics of Philippine Stichopus cf. horrens reveals diverse classes of valuable small molecules for biomedical applications
Source: PLoS One. 2023 Dec 6;18(12):e0294535. doi: 10.1371/journal.pone.0294535 (PMC10699614; doi:10.1371/journal.pone.0294535)
Supplement: S2 Table — (DOCX) [file pone.0294535.s007.docx]

**S2 Table. List of putatively identified fatty acids from *S. cf. horrens*.**

|  | **Compound Name** | **tR**  **(mins.)** | **Major**  **Ion** | **Experimental**  **Mass** | **Theoretical**  **Mass** | **ppm**  **error** | **Cosine** | **Body Wall** | | | **Viscera** | | |
| --- | --- | --- | --- | --- | --- | --- | --- | --- | --- | --- | --- | --- | --- |
|  |  |  |  |  |  |  |  | **crude** | **iBOH** | **hex** | **crude** | **iBOH** | **hex** |
| 1 | 5(S)-hydroxyeicosatetraenoic acid | 4.74 | [M+H]+ | 303.2309 | 303.2319 | 3.17 | 0.83 |  |  |  |  |  |  |
| 2 | Linoleic Acid | 4.75 | [M+H]+ | 303.2329 | 303.2324 | 1.63 | 0.83 |  |  |  |  |  |  |
| 3 | cis-5,8,11,14-Eicosatetraenoic acid | 5.00 | [M+H]+ | 305.2462 | 305.2475 | 4.29 | 0.86 |  |  |  |  |  |  |
| 5 | Arachidonic Acid Methyl Ester | 5.19 | [M+H]+ | 319.2629 | 319.2632 | 0.81 | MN/FA |  |  |  |  |  |  |
| 4 | Arachidonic Acid | 5.01 | [M+H]+ | 305.2462 | 305.2475 | 4.29 | 0.83 |  |  |  |  |  |  |
| 6 | 9-Octadecenamide | 5.2 | [M+H]+ | 282.2796 | 282.2791 | 1.63 | 0.86 |  |  |  |  |  |  |
| 7 | cis-7,10,13,16,19-Docosapentaenoic acid | 5.57 | [M+H]+ | 331.2626 | 331.2632 | 1.69 | 0.83 |  |  |  |  |  |  |
| 8 | Stearamide | 5.65 | [M+H]+ | 284.2953 | 284.2948 | 1.79 | MN/FA |  |  |  |  |  |  |
| 9 | Arachidonic acid ethyl ester | 5.83 | [M+H]+ | 333.2799 | 333.2788 | 3.27 | 0.83 |  |  |  |  |  |  |
| 10 | Palmitamide | 6.08 | [M+H]+ | 256.2642 | 256.2635 | 2.77 | MN/FA |  |  |  |  |  |  |
