# Supplementary material for: Comprehensive metabolomics of Philippine Stichopus cf. horrens reveals diverse classes of valuable small molecules for biomedical applications
Source: PLoS One. 2023 Dec 6;18(12):e0294535. doi: 10.1371/journal.pone.0294535 (PMC10699614; doi:10.1371/journal.pone.0294535)
Supplement: S3 Table — (DOCX) [file pone.0294535.s008.docx]

**S3 Table. List of putatively identified acylcarnitines from *S. cf. horrens*.**

|  | **Compound Name** | **tR**  **(mins.)** | **Major**  **Ion** | **Experimental**  **Mass** | **Theoretical**  **Mass** | **ppm**  **error** | **Cosine** | **Body Wall** | | | **Viscera** | | |
| --- | --- | --- | --- | --- | --- | --- | --- | --- | --- | --- | --- | --- | --- |
|  |  |  |  |  |  |  |  | **crude** | **iBOH** | **hex** | **crude** | **iBOH** | **hex** |
| 1 | Myristoylcarnitine | 2.72 | [M+H]+ | 372.3113 | 372.3108 | 1.24 | MN/FA |  |  |  |  |  |  |
| 2 | Palmitoleoylcarnitine | 2.79 | [M+H]+ | 398.3255 | 398.3265 | 2.49 | MN/FA |  |  |  |  |  |  |
| 3 | Acylcarnitine(15:0) | 2.82 | [M+H]+ | 386.3258 | 386.3265 | 1.79 | MN/FA |  |  |  |  |  |  |
| 4 | Arachidonoylcarnitine | 2.88 | [M+H]+ | 448.3414 | 448.3421 | 1.65 | MN/FA |  |  |  |  |  |  |
| 5 | Palmitoylcarnitine | 2.96 | [M+H]+ | 400.344 | 400.3421 | 4.65 | MN/FA |  |  |  |  |  |  |
| 6 | Stearoylcarnitine | 2.98 | [M+H]+ | 428.3731 | 428.3734 | 0.79 | MN/FA |  |  |  |  |  |  |
| 7 | Oleoylcarnitine | 3.01 | [M+H]+ | 426.3573 | 426.3578 | 1.15 | MN/FA |  |  |  |  |  |  |
