# Supplementary material for: Comprehensive metabolomics of Philippine Stichopus cf. horrens reveals diverse classes of valuable small molecules for biomedical applications
Source: PLoS One. 2023 Dec 6;18(12):e0294535. doi: 10.1371/journal.pone.0294535 (PMC10699614; doi:10.1371/journal.pone.0294535)
Supplement: S4 Table — (DOCX) [file pone.0294535.s009.docx]

**Table S4. List of putatively identified sphingolipids from S. cf. horrens.**

|  | **Compound Name** | **tR**  **(mins.)** | **Major**  **Ion** | **Experimental**  **Mass** | **Theoretical**  **Mass** | **ppm**  **error** | **Cosine** | **Body Wall** | | | **Viscera** | | |
| --- | --- | --- | --- | --- | --- | --- | --- | --- | --- | --- | --- | --- | --- |
|  |  |  |  |  |  |  |  | **crude** | **iBOH** | **hex** | **crude** | **iBOH** | **hex** |
| 1 | GalCer(d18:3/18:1) | 5.66 | [M+H]+ | 722.5538 | 722.5565 | 3.79 | MN/FA |  |  |  |  |  |  |
| 2 | GalCer(d19:1/18:1) | 5.67 | [M+H]+ | 740.6003 | 740.6035 | 4.31 | MN/FA |  |  |  |  |  |  |
| 3 | PC(18:3/18:4) | 5.79 | [M+H]+ | 776.5231 | 776.5225 | 0.80 | MN/FA |  |  |  |  |  |  |
| 4 | GlcCer(d19:3 /18:0 OH) | 5.86 | [M+H]+ | 754.5851 | 754.5873 | 2.86 | MN/FA |  |  |  |  |  |  |
| 5 | GalCer(d18:3/18:0) | 5.89 | [M+H]+ | 724.5721 | 724.5722 | 0.12 | MN/FA |  |  |  |  |  |  |
| 6 | GalCer(d18:2/18:0) | 5.89 | [M+H]+ | 726.5853 | 726.5878 | 3.50 | MN/FA |  |  |  |  |  |  |
| 7 | GlcCer(d19:3/18:1) | 5.92 | [M+H]+ | 736.5736 | 736.5722 | 1.91 | MN/FA |  |  |  |  |  |  |
| 8 | GlcCer(d19:3/18:0) | 5.95 | [M+H]+ | 738.5860 | 738.5878 | 2.49 | MN/FA |  |  |  |  |  |  |
| 9 | GlcCer(d17:0/24:1 OH) | 6.23 | [M+H]+ | 830.6682 | 830.6716 | 4.06 | MN/FA |  |  |  |  |  |  |
| 10 | HexCer(d18:0/ 21:3) | 6.24 | [M+H]+ | 766.6166 | 766.6192 | 3.33 | MN/FA |  |  |  |  |  |  |
| 11 | HexCer(d17:0/18:0 OH) | 6.25 | [M+H]+ | 748.5942 | 748.5933 | 1.18 | MN/FA |  |  |  |  |  |  |
| 12 | GlcCer(d16:1/24:2) | 6.3 | [M+H]+ | 780.6377 | 780.6348 | 3.71 | MN/FA |  |  |  |  |  |  |
| 13 | HexCer(d16:1/24:1 OH) | 6.4 | [M+H]+ | 798.6464 | 798.6454 | 1.30 | MN/FA |  |  |  |  |  |  |
| 14 | HexCer 36:3, O3 | 6.43 | [M+HCOO]- | 784.5557 | 784.5575 | 2.29 | MN/FA |  |  |  |  |  |  |
| 15 | HexCer(d18:3/24:2) | 6.44 | [M+H]+ | 804.6365 | 804.6348 | 2.11 | MN/FA |  |  |  |  |  |  |
| 16 | HexCer(d18:2/24:2) | 6.44 | [M+H]+ | 806.6517 | 806.6505 | 1.55 | MN/FA |  |  |  |  |  |  |
| 17 | GlcCer(d17:1 /24:2) | 6.45 | [M+H]+ | 794.6486 | 794.6505 | 2.33 | MN/FA |  |  |  |  |  |  |
| 18 | GalCer(d18:3 /24:1 OH) | 6.46 | [M+H]+ | 822.6470 | 822.6454 | 1.99 | MN/FA |  |  |  |  |  |  |
| 19 | HexCer(d19:3/24:2) | 6.49 | [M+H]+ | 818.6495 | 818.6505 | 1.16 | MN/FA |  |  |  |  |  |  |
| 20 | GalCer(d17:1 /24:1 OH) | 6.52 | [M+H]+ | 812.6640 | 812.6610 | 3.68 | MN/FA |  |  |  |  |  |  |
| 21 | HexCer(d17:1/22:1) | 6.56 | [M+H]+ | 768.6320 | 768.6348 | 3.64 | MN/FA |  |  |  |  |  |  |
| 22 | GalCer(d18:3/18:0) | 6.71 | [M+HCOO]- | 768.5637 | 768.5631 | 0.75 | MN/FA |  |  |  |  |  |  |
| 23 | GlcCer(d19:3 /18:0 OH) | 6.72 | [M+HCOO]- | 798.5697 | 798.5737 | 5.00 | MN/FA |  |  |  |  |  |  |
| 24 | GlcCer(d18:2 /18:0 OH) | 6.73 | [M+HCOO]- | 786.5730 | 786.5737 | 0.88 | MN/FA |  |  |  |  |  |  |
| 25 | HexCer 39:3, O3 | 7.28 | [M+HCOO]- | 826.6050 | 826.6044 | 0.73 | MN/FA |  |  |  |  |  |  |
| 26 | HexCer 40:1, O4 | 7.29 | [M+HCOO]- | 860.6420 | 860.6463 | 5.00 | MN/FA |  |  |  |  |  |  |
| 27 | HexCer 41:4, O3 | 7.41 | [M+HCOO]- | 852.6169 | 852.6201 | 3.75 | MN/FA |  |  |  |  |  |  |
| 28 | GlcCer(d17:0/24:1 OH) | 8.32 | [M+HCOO]- | 874.6597 | 874.6620 | 2.63 | MN/FA |  |  |  |  |  |  |
| 29 | HexCer 42:4, O3 | 8.34 | [M+HCOO]- | 866.6357 | 866.6357 | 0.00 | MN/FA |  |  |  |  |  |  |
| 30 | HexCer 40:2, O3 | 8.53 | [M+HCOO]- | 842.6339 | 842.6357 | 2.14 | MN/FA |  |  |  |  |  |  |
| 31 | GalCer(d17:1 /24:1 OH) | 8.61 | [M+HCOO]- | 856.6522 | 856.6514 | 0.93 | MN/FA |  |  |  |  |  |  |
| 32 | HexCer 43:4, O3 | 8.61 | [M+HCOO]- | 880.6473 | 880.6514 | 4.66 | MN/FA |  |  |  |  |  |  |
| 33 | HexCer 36:0, O6 | 8.63 | [M+HCOO]- | 838.5918 | 838.5892 | 3.10 | MN/FA |  |  |  |  |  |  |
| 34 | GlcCer(d19:2 /22:1 OH) | 8.7 | [M+HCOO]- | 854.6331 | 854.6363 | 3.73 | MN/FA |  |  |  |  |  |  |
| 35 | HexCer 39:1. O3 | 8.7 | [M+HCOO]- | 830.6330 | 830.6357 | 3.25 | MN/FA |  |  |  |  |  |  |
| 36 | HexCer 40:1, O3 | 8.81 | [M+HCOO]- | 844.6472 | 844.6514 | 4.97 | MN/FA |  |  |  |  |  |  |
| 37 | HexCer 42:3;O2 | 9.15 | [M+HCOO]- | 868.6503 | 868.6514 | 1.27 | MN/FA |  |  |  |  |  |  |
| 38 | HexCer 41:0; O4 | 10.05 | [M+HCOO]- | 876.6776 | 876.6776 | 0.00 | MN/FA |  |  |  |  |  |  |
| 39 | HexCer 43:3; O3 | 10.05 | [M+HCOO]- | 882.6660 | 882.6670 | 1.13 | MN/FA |  |  |  |  |  |  |

****Gal – Galactose, Glc – Glucose, Hex – Hexose, Cer - Ceramide***
