# Supplementary material for: Comprehensive metabolomics of Philippine Stichopus cf. horrens reveals diverse classes of valuable small molecules for biomedical applications
Source: PLoS One. 2023 Dec 6;18(12):e0294535. doi: 10.1371/journal.pone.0294535 (PMC10699614; doi:10.1371/journal.pone.0294535)
Supplement: S5 Table — (DOCX) [file pone.0294535.s010.docx]

**S5 Table. List of putatively identified phosphatidylinositols from *S. cf. horrens*.**

|  | **Compound Name** | **tR**  **(mins.)** | **Major**  **Ion** | **Experimental**  **Mass** | **Theoretical**  **Mass** | **ppm**  **error** | **Cosine** | **Body Wall** | | | **Viscera** | | |
| --- | --- | --- | --- | --- | --- | --- | --- | --- | --- | --- | --- | --- | --- |
|  |  |  |  |  |  |  |  | **crude** | **iBOH** | **hex** | **crude** | **iBOH** | **hex** |
| 1 | LysoPI(20:5) | 6.17 | [M-H]- | 617.2734 | 617.2732 | 0.26 | MN/FA |  |  |  |  |  |  |
| 2 | LysoPI(20:4) | 6.54 | [M-H]- | 619.2898 | 619.2889 | 1.47 | MN/FA |  |  |  |  |  |  |
| 3 | PI(O-21:4) | 6.82 | [M-H]- | 633.3055 | 633.3045 | 1.52 | MN/FA |  |  |  |  |  |  |
| 4 | PI(O-22:4) | 7.18 | [M-H]- | 647.3185 | 647.3202 | 2.61 | MN/FA |  |  |  |  |  |  |
| 5 | LPI(18:0) | 7.53 | [M-H]- | 599.3195 | 599.3196 | 0.17 | MN/FA |  |  |  |  |  |  |
| 6 | PI(O-18:0) | 7.79 | [M-H]- | 585.3403 | 585.3409 | 1.06 | MN/FA |  |  |  |  |  |  |
| 7 | LPI(O-21:0) | 7.8 | [M-H]- | 625.3329 | 625.3358 | 4.70 | MN/FA |  |  |  |  |  |  |
| 8 | LPI(19:0) | 7.88 | [M-H]- | 613.3369 | 613.3358 | 1.73 | MN/FA |  |  |  |  |  |  |
| 9 | LPI(21:1) | 8.15 | [M-H]- | 639.3492 | 639.3515 | 3.58 | MN/FA |  |  |  |  |  |  |
| 10 | LPI(20:0) | 8.34 | [M-H]- | 627.3521 | 627.3515 | 0.97 | MN/FA |  |  |  |  |  |  |
| 11 | LPI(22:1) | 8.58 | [M-H]- | 653.3663 | 653.3671 | 1.29 | MN/FA |  |  |  |  |  |  |
| 12 | LPI(21:0) | 8.79 | [M-H]- | 641.3649 | 641.3671 | 3.49 | MN/FA |  |  |  |  |  |  |
| 13 | LPI(23:1) | 8.97 | [M-H]- | 667.3820 | 667.3828 | 1.18 | MN/FA |  |  |  |  |  |  |
| 14 | LPI(22:0) | 9.33 | [M-H]- | 655.3804 | 655.3828 | 3.65 | MN/FA |  |  |  |  |  |  |

****LPI - Lysophosphatidylinositol***
