# Supplementary material for: Comprehensive metabolomics of Philippine Stichopus cf. horrens reveals diverse classes of valuable small molecules for biomedical applications
Source: PLoS One. 2023 Dec 6;18(12):e0294535. doi: 10.1371/journal.pone.0294535 (PMC10699614; doi:10.1371/journal.pone.0294535)
Supplement: S6 Table — (DOCX) [file pone.0294535.s011.docx]

**S6 Table. List of putatively identified phosphatidylserines from *S. cf. horrens*.**

|  | **Compound Name** | **tR**  **(mins.)** | **Major**  **Ion** | **Experimental**  **Mass** | **Theoretical**  **Mass** | **ppm**  **error** | **Cosine** | **Body Wall** | | | **Viscera** | | |
| --- | --- | --- | --- | --- | --- | --- | --- | --- | --- | --- | --- | --- | --- |
|  |  |  |  |  |  |  |  | **crude** | **iBOH** | **hex** | **crude** | **iBOH** | **hex** |
| 1 | LPS 18:0 | 7.23 | [M-H]- | 524.2997 | 524.2988 | 1.72 | MN/FA |  |  |  |  |  |  |
| 2 | LPS O-18:0 | 7.7 | [M-H]- | 510.3183 | 510.32013 | 3.59 | MN/FA |  |  |  |  |  |  |
| 3 | PS(O-20:1) | 7.74 | [M-H]- | 550.314 | 550.31504 | 1.89 | MN/FA |  |  |  |  |  |  |
| 4 | PS(O-21:1) | 8.05 | [M-H]- | 564.3282 | 564.33069 | 4.41 | MN/FA |  |  |  |  |  |  |
| 5 | PS(O-22:1) | 8.5 | [M-H]- | 578.3461 | 578.34634 | 0.41 | MN/FA |  |  |  |  |  |  |
| 6 | PS(23:1) | 8.82 | [M-H]- | 592.3616 | 592.36199 | 0.66 | MN/FA |  |  |  |  |  |  |

***LPS - *Lysophosphatidylserine***
