# Supplementary material for: Comprehensive metabolomics of Philippine Stichopus cf. horrens reveals diverse classes of valuable small molecules for biomedical applications
Source: PLoS One. 2023 Dec 6;18(12):e0294535. doi: 10.1371/journal.pone.0294535 (PMC10699614; doi:10.1371/journal.pone.0294535)
Supplement: S7 Table — (DOCX) [file pone.0294535.s012.docx]

**S7 Table. List of putatively identified phosphatidylethanolamines from *S. cf. horrens*.**

|  | **Compound Name** | **tR**  **(mins.)** | **Major**  **Ion** | **Experimental**  **Mass** | **Theoretical**  **Mass** | **ppm**  **error** | **Cosine** | **Body Wall** | | | **Viscera** | | |
| --- | --- | --- | --- | --- | --- | --- | --- | --- | --- | --- | --- | --- | --- |
|  |  |  |  |  |  |  |  | **crude** | **iBOH** | **hex** | **crude** | **iBOH** | **hex** |
| 1 | PE(20:4) | 3.52 | [M+H]+ | 502.2907 | 502.2928 | 4.22 | MN/FA |  |  |  |  |  |  |
| 2 | PE(20:5) | 3.52 | [M+H]+ | 500.2787 | 500.2772 | 3.06 | MN/FA |  |  |  |  |  |  |
| 3 | PE(O-20:5) | 3.57 | [M-H]- | 498.2613 | 498.2620 | 1.34 | MN/FA |  |  |  |  |  |  |
| 4 | LPE(21:4) | 3.71 | [M+H]+ | 516.3091 | 516.3090 | 0.19 | MN/FA |  |  |  |  |  |  |
| 5 | PE(O-20:4) | 3.73 | [M-H]- | 500.2791 | 500.2772 | 3.86 | MN/FA |  |  |  |  |  |  |
| 6 | LPE(P18:0) | 3.83 | [M-H]- | 464.3132 | 464.3147 | 3.12 | MN/FA |  |  |  |  |  |  |
| 7 | PE(O-21:4) | 3.96 | [M-H]- | 514.295 | 514.2934 | 3.11 | MN/FA |  |  |  |  |  |  |
| 8 | PE(O-22:5) | 4.04 | [M-H]- | 526.2943 | 526.2934 | 1.71 | MN/FA |  |  |  |  |  |  |
| 9 | LPE(P18:1) | 4.16 | [M-H]- | 462.2989 | 462.2990 | 0.22 | MN/FA |  |  |  |  |  |  |
| 10 | PE(O-18:0) | 4.41 | [M-H]- | 466.3274 | 466.3298 | 5.00 | MN/FA |  |  |  |  |  |  |
| 11 | PE(21:2) | 4.44 | [M-H]- | 532.3019 | 532.3039 | 3.76 | MN/FA |  |  |  |  |  |  |
| 12 | PE(O18:1) | 4.46 | [M-H]- | 403.2601 | 403.2619 | 4.14 | MN/FA |  |  |  |  |  |  |
| 13 | LPE(P19:1) | 4.65 | [M-H]- | 478.3298 | 478.3308 | 2.09 | MN/FA |  |  |  |  |  |  |
| 14 | LPE(P19:0) | 4.65 | [M-H]- | 480.3473 | 480.3465 | 1.77 | MN/FA |  |  |  |  |  |  |
| 15 | PE(O-20:1) | 4.7 | [M-H]- | 520.3404 | 520.3403 | 0.16 | MN/FA |  |  |  |  |  |  |
| 16 | PE(O-19:0) | 4.84 | [M-H]- | 496.3388 | 496.3409 | 4.15 | MN/FA |  |  |  |  |  |  |
| 17 | PE(22:1) | 4.88 | [M-H]- | 534.3533 | 534.35595 | 4.96 | MN/FA |  |  |  |  |  |  |
| 18 | :PE(O-20:1) | 5.03 | [M-H]- | 492.3459 | 492.3454 | 1.01 | MN/FA |  |  |  |  |  |  |
| 19 | PE(O-20:0) | 5.05 | [M-H]- | 522.3556 | 522.3560 | 0.70 | MN/FA |  |  |  |  |  |  |
| 20 | PE(23:1) | 5.15 | [M-H]- | 548.3702 | 548.3716 | 2.55 | MN/FA |  |  |  |  |  |  |
| 21 | PE(22:0) | 5.3 | [M-H]- | 536.3718 | 536.3716 | 0.37 | MN/FA |  |  |  |  |  |  |
| 22 | 1-Oleoyl-2-acetyl-sn-glycerol | 5.81 | [M+H]+ | 381.3010 | 381.3005 | 1.31 | 0.77 |  |  |  |  |  |  |
| 23 | PE(P-18:0/20:5) | 6.58 | [M+H]+ | 750.5448 | 750.5432 | 2.11 | MN/FA |  |  |  |  |  |  |
| 24 | PE(P-18:0/20:4) | 6.91 | [M+H]+ | 752.5585 | 752.5594 | 1.20 | 0.88 |  |  |  |  |  |  |
| 25 | PE(P-18:0/22:4) | 8.51 | [M-H]- | 778.5761 | 778.5751 | 1.28 | MN/FA |  |  |  |  |  |  |
| 26 | PE(P-18:0/22:4) | 8.51 | [M-H]- | 778.5761 | 778.5751 | 1.28 | MN/FA |  |  |  |  |  |  |
| 27 | PE 43:4 | 8.61 | [M-H]- | 838.5938 | 838.5962 | 2.86 | MN/FA |  |  |  |  |  |  |
| 28 | PE(P-18:0/20:4) | 8.88 | [M-H]- | 750.5441 | 750.5443 | 0.28 | MN/FA |  |  |  |  |  |  |
| 29 | PE 42:3 OH | 9.6 | [M-H]- | 840.6111 | 840.6118 | 0.83 | MN/FA |  |  |  |  |  |  |
| 30 | PE(P-20:1/20:4) | 9.88 | [M-H]- | 764.561 | 764.5594 | 2.09 | MN/FA |  |  |  |  |  |  |
| 31 | PE(P-18:0/22:3) | 10.1 | [M-H]- | 780.5887 | 780.5913 | 3.28 | MN/FA |  |  |  |  |  |  |
| 32 | PE(P-18:0/22:3) | 10.1 | [M-H]- | 780.5887 | 780.5913 | 3.28 | MN/FA |  |  |  |  |  |  |

****LPE - Lysophosphatidylethanolamine***
