# Supplementary material for: Comprehensive metabolomics of Philippine Stichopus cf. horrens reveals diverse classes of valuable small molecules for biomedical applications
Source: PLoS One. 2023 Dec 6;18(12):e0294535. doi: 10.1371/journal.pone.0294535 (PMC10699614; doi:10.1371/journal.pone.0294535)
Supplement: S8 Table — (DOCX) [file pone.0294535.s013.docx]

**S8 Table. List of putatively identified phosphatidylcholines from *S. cf. horrens***

|  | **Compound Name** | **tR (mins.)** | **Major Ion** | **Experimental Mass** | **Theoretical Mass** | **ppm error** | **Cosine** | **Body Wall** | | | **Viscera** | | |
| --- | --- | --- | --- | --- | --- | --- | --- | --- | --- | --- | --- | --- | --- |
|  |  |  |  |  |  |  |  | **crude** | **iBOH** | **hex** | **crude** | **iBOH** | **hex** |
| 1 | PC(20:4) | 3.91 | [M+HCOO]- | 558.3316 | 558.3307 | 1.63 | MN/FA |  |  |  |  |  |  |
| 2 | PC(O-18:1) | 4.28 | [M+HCOO]- | 552.3671 | 552.3671 | 0.04 | MN/FA |  |  |  |  |  |  |
| 3 | PC(O-13:0) | 3.66 | [M-H]- | 438.2919 | 438.2900 | 4.34 | MN/FA |  |  |  |  |  |  |
| 4 | PC(O-15:0) | 4.19 | [M-H]- | 466.3318 | 466.3303 | 3.22 | MN/FA |  |  |  |  |  |  |
| 5 | PC(O-17:1) | 4.29 | [M-H]- | 492.3459 | 492.3460 | 0.10 | MN/FA |  |  |  |  |  |  |
| 6 | PC(O-17:0) | 4.73 | [M-H]- | 494.3608 | 494.3616 | 1.62 | MN/FA |  |  |  |  |  |  |
| 7 | PC(O-16:1) | 3.82 | [M+HCOO]- | 524.3372 | 524.3358 | 2.71 | MN/FA |  |  |  |  |  |  |
| 8 | PC(P-19:5) | 3.91 | [M-H]- | 528.3069 | 528.3090 | 4.01 | MN/FA |  |  |  |  |  |  |
| 9 | PC(O-18:1) | 4.09 | [M+HCOO]- | 550.3525 | 550.3514 | 1.94 | MN/FA |  |  |  |  |  |  |
| 10 | PC(O-18:0) | 4.68 | [M+HCOO]- | 554.3807 | 554.3827 | 3.61 | MN/FA |  |  |  |  |  |  |
| 11 | PC(O-17:0;O) | 3.91 | [M+HCOO]- | 556.3593 | 556.3620 | 4.83 | MN/FA |  |  |  |  |  |  |
| 12 | PC(O-18:2;O) | 4.21 | [M+HCOO]- | 566.3469 | 566.3458 | 1.94 | MN/FA |  |  |  |  |  |  |
| 13 | PC(O-18:1;O) | 4.57 | [M+HCOO]- | 568.3632 | 568.3615 | 3.08 | MN/FA |  |  |  |  |  |  |
| 14 | PC(O-20:5) | 4.04 | [M+HCOO]- | 527.3342 | 527.3352 | 1.95 | MN/FA |  |  |  |  |  |  |
| 15 | PC(O-20:1) | 4.91 | [M+HCOO]- | 580.3966 | 580.3978 | 2.11 | MN/FA |  |  |  |  |  |  |
| 16 | PC(O-20:0) | 5.28 | [M+HCOO]- | 582.4111 | 582.4135 | 4.08 | MN/FA |  |  |  |  |  |  |
| 17 | LPC(20:5) | 3.73 | [M+HCOO]- | 586.3118 | 586.314492 | 4.59 | MN/FA |  |  |  |  |  |  |
| 18 | PC(O-23:1) | 5.47 | [M+HCOO]- | 636.4219 | 636.424042 | 3.37 | MN/FA |  |  |  |  |  |  |
| 19 | PC(20:1) | 4.73 | [M+HCOO]- | 594.3768 | 594.37764 | 1.41 | MN/FA |  |  |  |  |  |  |
| 20 | PC(20:0) | 5.13 | [M+HCOO]- | 596.3905 | 596.39329 | 4.68 | MN/FA |  |  |  |  |  |  |
| 21 | PC(O-21:1) | 5 | [M+HCOO]- | 608.3922 | 608.392742 | 0.89 | MN/FA |  |  |  |  |  |  |
| 22 | LPC(22:1) | 5.19 | [M+HCOO]- | 622.4073 | 622.408392 | 1.75 | MN/FA |  |  |  |  |  |  |
| 23 | LPC(23:1) | 5.47 | [M+HCOO]- | 636.4219 | 636.424042 | 3.37 | MN/FA |  |  |  |  |  |  |
| 24 | LPC(22:1) | 5.46 | [M-H]- | 576.4026 | 576.402895 | 0.51 | MN/FA |  |  |  |  |  |  |
| 25 | PC(P-18:1/O-19:1) | 9.69 | [M+HCOO]- | 816.6092 | 816.6129 | 4.53 | MN/FA |  |  |  |  |  |  |
| 26 | PC(P-16:0/O-20:4) | 8.43 | [M+HCOO]- | 812.5824 | 812.58109 | 1.61 | MN/FA |  |  |  |  |  |  |
| 27 | PC(P-16:1/O-20:4) | 7.98 | [M+HCOO]- | 810.564 | 810.56544 | 1.78 | MN/FA |  |  |  |  |  |  |
| 28 | PC(P-18:0/20:4) | 8.53 | [M+HCOO]- | 838.5997 | 838.59674 | 3.53 | MN/FA |  |  |  |  |  |  |
| 29 | PC(P-18:0/20:5) | 8.23 | [M+HCOO]- | 836.5787 | 836.58109 | 2.86 | MN/FA |  |  |  |  |  |  |
| 30 | PC(P-19:0/20:4) | 10.23 | [M+HCOO]- | 854.6271 | 854.62804 | 1.10 | MN/FA |  |  |  |  |  |  |
| 31 | PC O-36:5, O | 8.11 | [M+HCOO]- | 826.5638 | 826.5598 | 4.84 | MN/FA |  |  |  |  |  |  |
| 32 | PC O-37:5 | 8.3 | [M+HCOO]- | 824.581 | 824.5805 | 0.61 | MN/FA |  |  |  |  |  |  |
| 33 | LPC(16:0) | 4.05 | [M+HCOO]- | 540.3295 | 540.3301 | 1.11 | MN/FA |  |  |  |  |  |  |
| 34 | PC O-38:4 | 9.72 | [M+HCOO]- | 840.6111 | 840.6118 | 0.83 | MN/FA |  |  |  |  |  |  |
| 35 | LPC 20:4 | 3.96 | [M+HCOO]- | 588.3316 | 588.3301 | 2.55 | MN/FA |  |  |  |  |  |  |
| 36 | LPC O-14:0 | 3.93 | [M-H]- | 452.3148 | 452.3141 | 1.55 | MN/FA |  |  |  |  |  |  |

****LPC - Lysophosphatidylcholine***
