# Supplementary material for: Comprehensive metabolomics of Philippine Stichopus cf. horrens reveals diverse classes of valuable small molecules for biomedical applications
Source: PLoS One. 2023 Dec 6;18(12):e0294535. doi: 10.1371/journal.pone.0294535 (PMC10699614; doi:10.1371/journal.pone.0294535)
Supplement: S9 Table — (DOCX) [file pone.0294535.s014.docx]

**S9 Table. Data preprocessing workflow and parameters used for feature-based and ion-identity molecular networking via MZMine.**

| **Mass Detection** | |
| --- | --- |
| MS1 Filter | 1.0E4 |
| MS2 Filter | 1.0E4 |
| Polarity | Any |
| Spectrum Type | Centroid |
| **Peak Detection – ADAP Chromatogram Builder** | |
| Min. group size in 3 scans | 3 |
| Group Intensity Threshold | 1.0E4 |
| Min. Highest Intensity | 1.0E2 |
| m/z tolerance | 0.05 Da or 30 ppm |
| **Chromatogram Deconvolution – Baseline Cutoff** | |
| Min. Peak Height | 3.0E4 |
| Baseline | 1.0E4 |
| m/z center calculation | MEDIAN |
| **Deisotoping – Isotopes Peak Grouper** | |
| m/z tolerance | 0.02 Da or 20 ppm |
| Retention Time Tolerance | 0.2 mins. |
| Maximum Charge | 2 |
| Representative Isotope | Most Intense |
| **Join Aligner** | |
| m/z tolerance | 0.02 Da or 20 ppm |
| m/z weight | 80 |
| Retention time tolerance | 0.2 mins. |
| Retention time weight | 20 |
| **Gap-Filling – Peak Finder** | |
| Intensity Tolerance | 95% |
| m/z tolerance | 0.01 Da or 10 ppm |
| Retention Time Tolerance | 0.2 mins. |
| **Peak Filtering** | |
| Only Peaks with MS2 Scans | Yes |
| Duplicate Peak Filter | 0.02 Da or 20 ppm |
| Retention Time Tolerance | 0.2 mins. |
| **Row Grouping – metacorrelate** | |
| Min. height | 3.0E4 |
| Noise Level | 1.0E2 |
| Min. feature shape correlation | 85% (PEARSON) |
| Feature Height Correlation | No |
| **Identification – Ion Molecular Networking** | |
| m/z tolerance | 0.01 Da or 10 ppm |
| Ion identification template | custom |
|  |  |
