# Supplementary material for: Comprehensive metabolomics of Philippine Stichopus cf. horrens reveals diverse classes of valuable small molecules for biomedical applications
Source: PLoS One. 2023 Dec 6;18(12):e0294535. doi: 10.1371/journal.pone.0294535 (PMC10699614; doi:10.1371/journal.pone.0294535)
Supplement: S10 Table — (DOCX) [file pone.0294535.s015.docx]

**S10 Table. List of precursor ions common between positive and negative mode analysis.**

|  | Positive Ion | Negative Ion | Positive, tR | Negative tR | delta mass |
| --- | --- | --- | --- | --- | --- |
| 1 | 752.57 | 750.542 | 9.237 | 9.29 | 2.029 |
| 2 | 752.58 | 750.543 | 8.975 | 8.839 | 2.032 |
| 3 | 752.57 | 750.543 | 8.752 | 8.839 | 2.023 |
| 4 | 510.39 | 508.376 | 4.967 | 5.058 | 2.017 |
| 5 | 782.63 | 780.587 | 10.181 | 10.073 | 2.043 |
| 6 | 348.08 | 346.059 | 1.242 | 1.25 | 2.021 |
| 7 | 846.64 | 844.647 | 8.946 | 8.768 | 1.995 |
| 8 | 766.59 | 764.557 | 9.02 | 9.115 | 2.033 |
| 9 | 832.61 | 830.632 | 8.854 | 8.648 | 1.981 |
| 10 | 846.64 | 844.647 | 8.828 | 9.035 | 1.994 |
| 11 | 858.65 | 856.647 | 8.197 | 8.428 | 2.004 |
| 12 | 858.62 | 856.626 | 7.87 | 7.928 | 1.995 |
| 13 | 566.34 | 564.332 | 7.787 | 8.014 | 2.013 |
| 14 | 512.33 | 510.316 | 7.575 | 7.719 | 2.017 |
| 15 | 812.61 | 810.574 | 7.622 | 7.793 | 2.04 |
| 16 | 552.33 | 550.314 | 7.592 | 7.841 | 2.015 |
| 17 | 552.32 | 550.316 | 7.239 | 7.22 | 2.007 |
| 18 | 784.59 | 782.575 | 7.187 | 6.991 | 2.015 |
| 19 | 800.64 | 798.59 | 7.104 | 7.153 | 2.055 |
| 20 | 800.65 | 798.573 | 6.885 | 6.65 | 2.073 |
| 21 | 413.28 | 411.255 | 6.539 | 6.6 | 2.022 |
| 22 | 800.63 | 798.573 | 6.545 | 6.65 | 2.056 |
| 23 | 621.31 | 619.291 | 6.377 | 6.488 | 2.015 |
| 24 | 546.29 | 544.268 | 6.103 | 6.128 | 2.019 |
| 25 | 788.57 | 786.534 | 6.098 | 6.109 | 2.034 |
| 26 | 347.29 | 345.278 | 6.04 | 6.13 | 2.016 |
| 27 | 806.57 | 804.535 | 5.877 | 5.873 | 2.032 |
| 28 | 546.28 | 544.268 | 5.947 | 6.128 | 2.017 |
| 29 | 333.28 | 331.262 | 5.732 | 5.88 | 2.015 |
| 30 | 845.41 | 843.357 | 5.785 | 5.824 | 2.057 |
| 31 | 845.43 | 843.357 | 5.618 | 5.824 | 2.071 |
| 32 | 776.56 | 774.523 | 5.847 | 5.986 | 2.032 |
| 33 | 319.27 | 317.241 | 5.632 | 5.665 | 2.03 |
| 34 | 331.26 | 329.249 | 5.55 | 5.686 | 2.013 |
| 35 | 678.5 | 676.49 | 5.647 | 5.782 | 2.012 |
| 36 | 538.41 | 536.372 | 5.262 | 5.156 | 2.034 |
| 37 | 774.53 | 772.515 | 5.419 | 5.648 | 2.013 |
| 38 | 305.25 | 303.231 | 5.241 | 5.425 | 2.016 |
| 39 | 524.4 | 522.356 | 5.003 | 5.089 | 2.048 |
| 40 | 536.4 | 534.361 | 4.862 | 4.931 | 2.041 |
| 41 | 494.38 | 492.346 | 4.918 | 5.117 | 2.037 |
| 42 | 496.37 | 494.361 | 4.705 | 4.789 | 2.007 |
| 43 | 524.41 | 522.356 | 4.891 | 5.089 | 2.051 |
| 44 | 496.37 | 494.334 | 4.325 | 4.387 | 2.039 |
| 45 | 510.39 | 508.341 | 4.587 | 4.738 | 2.05 |
| 46 | 480.34 | 478.329 | 4.602 | 4.781 | 2.016 |
| 47 | 466.33 | 464.319 | 4.347 | 4.464 | 2.009 |
| 48 | 530.35 | 528.307 | 4.153 | 3.954 | 2.042 |
| 49 | 558.36 | 556.357 | 4.074 | 3.935 | 1.998 |
| 50 | 540.35 | 538.313 | 3.836 | 3.706 | 2.035 |
| 51 | 526.39 | 524.33 | 3.971 | 3.874 | 2.059 |
| 52 | 482.35 | 480.307 | 4.08 | 4.178 | 2.041 |
| 53 | 508.37 | 506.325 | 4.149 | 4.279 | 2.046 |
| 54 | 496.34 | 494.329 | 3.949 | 4.051 | 2.015 |
| 55 | 516.31 | 514.29 | 3.967 | 3.981 | 2.024 |
| 56 | 552.39 | 550.349 | 3.933 | 4.152 | 2.043 |
| 57 | 528.34 | 526.288 | 3.893 | 3.729 | 2.047 |
| 58 | 516.32 | 514.29 | 3.82 | 3.981 | 2.029 |
| 59 | 466.32 | 464.302 | 3.623 | 3.804 | 2.015 |
| 60 | 335.17 | 333.149 | 3.586 | 3.759 | 2.025 |
| 61 | 502.3 | 500.279 | 3.694 | 3.757 | 2.021 |
| 62 | 500.29 | 498.275 | 3.456 | 3.598 | 2.011 |
| 63 | 466.32 | 464.279 | 3.518 | 3.536 | 2.043 |
| 64 | 353.23 | 351.218 | 3.299 | 3.261 | 2.01 |
| 65 | 528.34 | 526.282 | 3.261 | 3.324 | 2.061 |
| 66 | 508.35 | 506.312 | 2.822 | 2.693 | 2.04 |
