# Supplementary material for: Comprehensive metabolomics of Philippine Stichopus cf. horrens reveals diverse classes of valuable small molecules for biomedical applications
Source: PLoS One. 2023 Dec 6;18(12):e0294535. doi: 10.1371/journal.pone.0294535 (PMC10699614; doi:10.1371/journal.pone.0294535)
Supplement: Attachment — Submitted filename: response to reviewers_2_Torreno PLOS.pdf [file pone.0294535.s019.pdf]

THE MARINE SCIENCE INSTITUTE
COLLEGE OF SCIENCE
UNIVERSITY OF THE PHILIPPINES
Velasquez Street, Diliman, Quezon City, Philippines

Mail : U.P.-P.O. Box 1 Diliman
1101 Quezon City, Philippines
E-mail : msiadmin@msi.upd.edu.ph

Tels. : (63-2) 922-3959 Direct line
981-8500 loc 2903
Fax : (63-2) 924-7678

September 20, 2023

RESPONSE TO REVIEWER COMMENTS

Dear Reviewers:

Thank you very much for your insightful comments and suggestions. Please see below our response and action to all the reviewer comments we received. We do hope that the actions we took suffice.

Reviewer Comments (from email body)	Remarks/Action Taken
Reviewer #2<br>The comments have been addressed in a satisfactory manner. I would suggest that the upcoming study that the authors plan of doing should focus on the quantitative analysis of the PLs as well.	Quantitative analysis is planned for future projects. Thank you.
Reviewer #3<br>1. The font sizes and resolution of the following figures are required to be improved to make them legible:<br>Figure 2 axes labels<br>Fig 3A-numbers, Figure 3B and D labels in the pie chart and name of metabolites.<br>Fig 4. labels in the network diagrams are not legible. Please improve the resolution of the figure.<br>Figure 6 Chemical reaction schemes and chemical structures. Similar figures in the SI have very good resolution.<br><br>I recommend accepting the manuscript only after the above revisions.	There seems to be a problem in compiling the PDF for review. The figures we uploaded and available through the blue link (found in the upper right hand corner of the figure pages) are high resolution and have passed the figure resolution requirement of PLOS One. I am attaching the email exchange with PLOS One editors as proof.<br><br>Nevertheless, we have corrected the figures and increased the font sizes according to the comments.
Reviewer # 4<br>The manuscript has many other grammatical and formatting errors which are to be fixed. Manuscript needs proper revision before submission.<br><br>Statistical significance of data is lacking.	It would be helpful if the reviewer points out example pages and lines that pertain to said errors. Nevertheless, we have reviewed the manuscript and found no significant grammatical nor formatting errors.

Figures are not clear.	As for the requirement for statistical significance of the data presented, we would like to highlight that the focus of the paper is the identification of metabolites from *S. horrens*.. The data analysis programs we used (e.g.,GNPS) have built-in metrics for statistical significance and confidence of mass assignments. Further, the goal of the study is to annotate a baseline metabolome of the species, which may serve as reference for future comparative and quantitative analyses.<br><br>Figures have been addressed in accordance with the comments of the Reviewers from the first round of review. There seems to be a problem in compiling the PDF for review. The figures we uploaded and available through the blue link (found in the upper right hand corner of the figure pages) are high resolution and have passed the resolution requirement of PLOS One. Nevertheless, we have corrected the figures and increased the font sizes according to the comments. We would like to humbly request the reviewers to download the files through the blue link provided to properly scale resolution, should the figure in the pdf still come out as blurry.
Reviewer Comments (from the attachment)	
1. The present study reveals the metabolite profile of S.cf. horrens using bioinformatics tools. The manuscript satisfies the current title but the overall reflection of importance of molecules for lacking. Addition of more literature regarding the relevance of the study will be better. It requires more clear view.	Thank you for this suggestion. We did include the relevance of our study in the introduction section (page 3 lines 6-9, 12-14) and in the discussion section (page 18 lines 12-14, page 19 lines 22-23, page 20 lines 2-3, 11-17, page 21 lines 5-13, 17-20, page 23 lines 8-12). But briefly, *S. horrens* is a high-value sea cucumber commodity in Asia and this species is the source of “gamat”, the wound-healing medicinal remedy. Unfortunately, even given its widespread use, there is no comprehensive metabolome reported for this species and possible attribution for the bioactivity of the sea cucumber extract. This study represents a baseline analysis of this sea cucumber metabolome. As for the molecules we identified, we have included literature references to support the bioactivity of the reported molecules.
2. In Materials and methods page 5, under heading Sampling and extraction of secondary metabolites from S.cf. horrens; age of ten S.cf.horrens is not mentioned	We did not indicate the age of the samples as we used broodstock animals for the study. We revise the methodology to include that the animals were mature adults. The metabolome

and also, whether sampling was done in triplicate or not.	we report is from a total of ten animals collected from the same hatchery.
3. In Materials and method page 7, under heading Bioinformatics analysis; Data was pre-processed using MZmine v2.52 should be Data was pre-processed using MZmine 2.52 similarly for Cytoscape too.	We have revised the manuscript to read MZmine 2.52 and Cytoscape 3.8.2. Thanks.
4. In Materials and method page 8, under heading Quantification of sulfated compounds; model number for the Shimadzu Multi-scanner needs to be reported.	Thank you for this. We have revised the manuscript to reflect the model and make of the multiscanner.
5. All the figures are of low quality. unpublishable quality is lacking. No conclusion can be drawn from the figures. The figure legends need to be rewritten. The legends should be succinct.	Please see the explanation (previous page). These have been addressed accordingly.
6. Figure 3 lacks the color scale which indicate which color corresponds to what. Although it is written in legend, but figures need to be self-explanatory.	These have been addressed accordingly.
7. Page 14, legend for figure 6 is to be removed. Page 16 again has legend for Fig6.	These have been corrected accordingly.
8. All the chemical formulas in Page 14-16 needs to have numerals as subscript.	These have been corrected accordingly.
9. Statistical significance of data is lacking in all figure legends.	This has been addressed earlier. Please see our remarks on Reviewer 4 comments found in previous pages.
10. The manuscript has many other grammatical and formatting errors which are to be fixed. Manuscript needs proper revision before submission.	This has been addressed earlier, please see remarks on comments on reviewer 4 comments in the previous page.

Again thank you very much.

Very truly yours,

Eizadora T. Yu, PhD
Professor
