# Supplementary material for: Comprehensive metabolomics of Philippine Stichopus cf. horrens reveals diverse classes of valuable small molecules for biomedical applications
Source: PLoS One. 2023 Dec 6;18(12):e0294535. doi: 10.1371/journal.pone.0294535 (PMC10699614; doi:10.1371/journal.pone.0294535)
Supplement: Attachment — Submitted filename: Reviewer-comment-sea-c.docx [file pone.0294535.s016.docx]

**Reviewer’s comments**

The authors in the present manuscript have investigated and characterized the metabolite profile of *S. cf. horrens* using various experimental and bioinformatics tools. Although the manuscript is well written, the overall motivation is lacking. *S. cf. horrens* is an important bioactive molecule, but there is not enough literature supporting this. The authors need to revise the manuscript to highlight the significance with some evidence. Is this the first study to characterize the *S. cf. horrens?*

The figures throughout the manuscript are of very poor quality and it is hard to access the conclusion drawn from the manuscript. The authors decode the presence of various phospholipids in the species, it would be helpful if they can provide the chemical structures as well. Also, the ratio of different types os phospholipids is not mentioned in the manuscript, but this could be a valuable information to access bioactivity and toxicity of the species. Certain abbrevaitions need full forms such as PUFAs, MS2LDA…etc.

Overall the manuscript needs significant revision and improvement before it is published.
