# Supplementary material for: Comprehensive metabolomics of Philippine Stichopus cf. horrens reveals diverse classes of valuable small molecules for biomedical applications
Source: PLoS One. 2023 Dec 6;18(12):e0294535. doi: 10.1371/journal.pone.0294535 (PMC10699614; doi:10.1371/journal.pone.0294535)
Supplement: Attachment — Submitted filename: The present study reveals the metabolite profile of S.docx [file pone.0294535.s018.docx]

1. The present study reveals the metabolite profile of *S.cf. horrens* using bioinformatics tools. The manuscript satisfies the current title but the overall reflection of importance of molecules for lacking. Addition of more literature regarding relevance of the study will be better. It requires more clear view.
2. In Materials and methods page 5, under heading Sampling and extraction of secondary metabolites from *S.cf. horrens*; age of ten *S.cf.horrens* is not mentioned and also, whether sampling was done in triplicate or not.
3. In Materials and method page 7, under heading Bioinformatics analysis; Data was pre-processed using MZmine v2.52 should be Data was pre-processed using MZmine 2.52 similarly for cytoscape too.
4. In Materials and method page 8, under heading Quantification of sulfated compouds; model number for the Shimadzu Multi-scanner needs to be reported.
5. All the figures are of low quality. Publishable quality is lacking. No conclusion can be drawn from the figures. The figure legends need to be rewritten. The legends should be succinct.
6. Figure 3 lacks the color scale which indicate which color corresponds to what. Although it is written in legend, but figures need to be self-explanatory.
7. Page 14, legend for figure 6 is to be removed. Page 16 again has legend for Fig 6.
8. All the chemical formulas in Page 14-16 needs to have numerals as subscript.
9. Statistical significance of data is lacking in all figure legends.
10. The manuscript has many other grammatical and formatting errors which are to be fixed. Manuscript needs proper revision before submission.
