# Supplementary material for: Comprehensive metabolomics of Philippine Stichopus cf. horrens reveals diverse classes of valuable small molecules for biomedical applications
Source: PLoS One. 2023 Dec 6;18(12):e0294535. doi: 10.1371/journal.pone.0294535 (PMC10699614; doi:10.1371/journal.pone.0294535)
Supplement: Attachment — Submitted filename: PONE-D-23-1172_response to reviewer comments.pdf [file pone.0294535.s017.pdf]

THE MARINE SCIENCE INSTITUTE

COLLEGE OF SCIENCE
UNIVERSITY OF THE PHILIPPINES
Velasquez Street, Diliman, Quezon City, Philippines

Mail : U.P.-P.O. Box 1 Diliman
1101 Quezon City, Philippines
E-mail : msiadmin@msi.upd.edu.ph

Tels. : (63-2) 922-3959 Direct line
981-8500 loc 2903
Fax : (63-2) 924-7678

RESPONSE TO REVIEWER COMMENTS

Dear Reviewers:

Thank you very much for your insightful comments and suggestions. Please see below our response and action to all the reviewer comments we received.

Reviewer Comments	Remarks/Action Taken
Reviewer #1<br>This study aimed to investigate the metabolite profile of Stichopus cf. horrens, a valuable sea cucumber species in Southeast Asia with medicinal properties and identify the compounds responsible for its bioactivities. Through LC-MS/MS experiments and open-access platforms such as GNPS, XCMS, and metaboAnalyst, the study identified various phospholipids, terpenoid glycosides, saponins, sulfated alkanes, and sterols in the crude samples. The study also revealed the abundance and distribution of these compounds in different body parts of the species, suggesting their potential ecological and morphological implications. The presence of terpenoid glycosides and saponins with reported anti-cancer benefits in the body wall was particularly noteworthy. The study provides a useful baseline for further comparative and exploratory studies and highlights the untapped potential of S. cf. horrens as a source of bioactive molecules. This study is novel as it represents the first biochemical analysis of the metabolite profile of S. cf. horrens and provides new insights into the potential bioactive compounds of this species.<br><br>1. The authors must remake figures 2-6. The figures are poor quality and items are not legible.	 <br>Figures have been redrawn as vector files and have been scaled accordingly.
Reviewer #2<br><br>1. The authors in the present manuscript have investigated and characterized the metabolite profile of S. cf. horrens using various experimental and bioinformatics tools. Although the manuscript is well written, the overall motivation is lacking. S. cf. horrens is an important bioactive molecule, but there is not enough literature supporting this. The authors need to revise the manuscript to highlight the significance with	 <br>Stichopus horrens is a particularly interesting organism as it is used as an ingredient for topical salves and exhibits a broad spectrum of body elasticity changes with varying stimuli. Much of the medicinal use of S. horrens in Southeast Asia is in the form of “gamat” oil or gels used primarily for wound healing. Although there is literature showing that S. horrens extracts facilitate wound healing,

some evidence. Is this the first study to characterize the S. cf. horrens?	very little known about what specific compounds are in the sea cucumber extracts. To our knowledge, this is the first study to perform a comprehensive structural characterization of metabolites extracted from S. cf. horrens. We have added this information in the introduction (Page 3, lines 9-10). We also added references to other biochemical studies on similar or related compounds (Page 20 lines 12-15; Page 21 lines 14-17; Page 23 lines 5-9) as additional evidence on their potential biomedical use.
2. The figures throughout the manuscript are of very poor quality and it is hard to access the conclusion drawn from the manuscript.	The figures have been redrawn and scaled accordingly as vector images.
3. The authors decode the presence of various phospholipids in the species, it would be helpful if they can provide the chemical structures as well.	The compound names we listed in the supplemental tables are conventional shorthand nomenclature of lipids. We have added footnotes in the tables as a guide to deducing structures from the shorthand notations.
4. Also, the ratio of different types os phospholipids is not mentioned in the manuscript, but this could be a valuable information to access bioactivity and toxicity of the species.	The focus of this study is the baseline characterization of the S. horrens metabolome. While we agree that it is valuable information, quantitation was not part of this study. However, we plan to quantify select PLs in the future.
5. Certain abbreviations need full forms such as PUFAs, MS2LDA…etc.	We have revised the manuscript to define the abbreviations listed, except for MS2LDA, which derives its name from the latent Dirichlet allocation algorithm but was not directly stated in the reference.
Overall the manuscript needs significant revision and improvement before it is published.	
Reviewer #3<br><br>All the figures, figure labels and figure legends are of low resolution and barely legible	The figures have been redrawn and scaled accordingly as vector images.
A discussion on how from literature one may assume that the compounds discovered here will have biomedical applications is warranted.	We have added references to other biochemical studies on the same or related compounds (Page 20 lines 12-15; Page 21 lines 14-17; Page 23 lines 5-9) as additional evidence on their potential biomedical use.

Respectfully yours,

Eizadora T. Yu
Corresponding author
